# Patients with sepsis exhibit mitochondrial biogenesis in peripheral blood immune cells

**DOI:** 10.1186/cc11716

**Published:** 2012-11-14

**Authors:** F Sjövall, S Morota, MJ Hansson, E Elmér

**Affiliations:** 1 Lund University, Lund, Sweden

## Background

Sepsis is one of the leading causes of admission to the ICU. After the initial proinflammatory response a gradual change towards a more anti-inflammatory pattern can be seen. In this latter stage, immune cell function has been suggested to be downregulated leading to an immunoparalysis, lending the patient more vulnerable to deleterious secondary infections. Mitochondrial dysfunction has been suggested to play a role in this immunoparalytic state and we therefore investigated mitochondrial respiratory function in peripheral blood immune cells (PBICs) in patients with sepsis and its evolvement over time.

## Methods

Twenty patients with severe sepsis or septic shock were included. PBICs were isolated from freshly drawn blood via density gradient centrifugation and analyzed three times during the first week after admission to the ICU (within 48 hours, days 3 to 4 and days 6 to 7). Mitochondrial respiration was examined with high-resolution respirometry in intact and permeabilized cells in order to evaluate both whole cell respiration as well as contribution of individual complexes. Mitochondrial DNA (mtDNA), cytochrome *c *(Cyt *c*) and citrate synthase (CS) were measured as indicators of change in cellular mitochondrial content.

## Results

In intact PBICs from septic patients, with endogenous substrates, there was a gradual increase in cellular respiration that was 73% higher after 1 week compared with controls (*P *= 0.003). In permeabilized cells, complex I, II and IV displayed increased respiration compared with controls already at days 1 to 2 (37%, 30% and 73% respectively, *P *< 0.001) and continued to increase to days 6 to 7 (68%, 68% and 108% respectively). The rise in mitochondrial respiration was paralleled by higher levels of CS activity and increased mtDNA and Cyt *c *content in cells from septic patients (90%, 143% and 231% for the respective parameter at days 6 to 7 compared with controls, *P *< 0.0001). Mortality for the septic patients was 25% by day 7. There was no difference in respiratory capacity between survivors and nonsurvivors at any of the time points measured.

## Conclusion

In PBICs from patients with severe sepsis or septic shock there is a gradual increase in mitochondrial respiratory capacity. This increase is probably due to mitochondrial biogenesis as indicated by increases in mitochondria-specific markers. Nonsurvivors displayed the same increase in respiration as survivors arguing against mitochondrial respiratory dysfunction and defect mitochondrial biogenesis, in PBICs, as a mediator of increased mortality in the septic condition.

